# Follow up assessment on the first local theranostic intra-cavitary Yttrium-90 citrate colloid irradiation for refractory cystic craniopharyngioma: Is it still a valuable treatment option?

**DOI:** 10.22038/aojnmb.2025.83694.1594

**Published:** 2025

**Authors:** Nadiah Abd Razak, Pung Choon Ping, Kamalia Kamarulzaman, Siti Zarina Amir Hassan

**Affiliations:** Department of Nuclear Medicine, Hospital Kuala Lumpur, Kuala Lumpur, Malaysia

**Keywords:** Cystic craniopharyngioma, Irradiation therapy, Yttrium-90, Follow up, Complication

## Abstract

Craniopharyngioma is a rare, benign intracranial tumour that can present with solid, cystic, or mixed solid-cystic characteristics. This case report aims to discuss the follow up assessment of our patient after one year of the irradiation therapy for craniopharyngioma. A 43-year-old male who has underlying refractory cystic craniopharyngioma requiring two-weekly aspirations, complicated with visual impairment and panhypopituitarism, was referred to our department for intra-cavitary irradiation therapy. Initial diagnostic assessment with Tc-99m MAA followed by dose calculation using the Backlund formula were conducted prior to the therapy. The patient received 6.22 mCi (230.14 MBq) of Ytrrium-90 citrate colloid via the Ommaya reservoir to deliver a radiation dose of 300 Gy to the tumour. Positive outcomes were observed as signified by the reduction on the aspirated cystic frequency and volume, tumour volume, improvement on the visual function and stable hormonal level. Despite the complications, the intra-cavitary irradiation therapy has demonstrated a significant and valuable therapeutic option for our patient in the management of refractory cystic craniopharyngioma.

## Introduction

 Craniopharyngioma is a rare benign intracranial tumour arising from the remnant of Rathke’s pouch that may present with solid, cystic, or mixed solid-cystic components (1). 

 The main treatment strategy for cranio-pharyngioma is surgical resection with or without radiotherapy (2, 3). However, in some patients, the anatomical location makes surgery or radiotherapy hazardous or unfeasible, resulting in exploration of alternative treatments, such as intra-cavitary irradiation therapy (2, 3). 

 Few studies of intra-cavitary irradiation therapy have demonstrated promising outcomes in treating patients with cystic craniopharyngioma with possible complications (4, 5). Our case report highlights the follow up assessment of the patient's condition, including the outcomes and complications observed one year after the irradiation therapy.

## Case report

**Figure 1:**
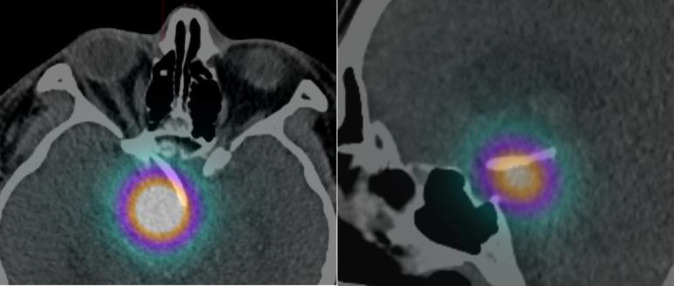
Post therapy Bremsstrahlung Yttrium-90 images using SPECT/CT. Tracer uptake was confined within the tumour with no evidence of leakage to the surrounding structures

 A 43-year-old male who has underlying refractory cystic craniopharyngioma requiring two-weekly aspirations, complicated with visual impairment and panhypopituitarism, was referred to our department for intra-cavitary irradiation therapy. Prior to the referral, he had twice cystic craniopharyngioma resection surgeries and right pterional craniotomy Ommaya shunt insertion. He was not suitable for radiotherapy as the craniopharyngioma mass was impinging onto the brainstem. The intracystic irradiation therapy was delivered with an initial diagnostic assessment using Tc-99m macro aggregated albumin (MAA). The required dose activity was calculated based on the Backlund formula. He subsequently received 6.22 mCi (230.14 MBq) of Yttrium-90 citrate colloid via the Ommaya reservoir with the intended radiation dose of 300 Gy to the tumour, estimated based on the pre-therapy Tc-99m MAA images. Post therapy, Bremsstrahlung Yttrium-90 single photon emission computed tomography/computed tomography (SPECT/CT) was acquired (Figure 1). Yttrium-90 positron emission tomography (PET) was not acquired due to inaccessibility of the PET machine in our center (6).

## Results

 The patient was evaluated in the nuclear medicine outpatient clinic at intervals of two to three months post-therapy. In addition to our follow up assessments, the patient attended multiple consultations with various specialities, including oncology, neurosurgery, endocrinology, and ophthalmology. During follow-up, evaluations were conducted based on cerebrospinal fluid (CSF) aspiration frequency and volume, tumour volume, visual function, and hormonal stability. 

 Imaging, such as contrast enhanced computed tomography (CECT) and magnetic resonance imaging (MRI) of the brain was performed at the third and eleventh months post therapy.

### CSF Aspiration

 Following intracystic irradiation therapy, the patient continued to require CSF aspirations via the Ommaya reservoir at the neurosurgical outpatient clinic. However, a significant reduction in the volume of aspirated CSF was observed as early as the first month post-therapy, with no further aspiration needed during the fifth and sixth months. Additionally, the frequency of CSF aspiration had markedly decreased from biweekly to approximately once per month (Table 1).

**Table 1:** Table of cerebrospinal fluid volume and culture & sensitivity aspirated through the Ommaya reservoir from June 2023 to February 2024 by the neurosurgical team

**Date**	**Cerebrospinal fluid volume (ml)**	**Cerebrospinal fluid culture and sensitivity**
26/06/2023	25	-
10/07/2023	30	Turbid; no growth after 48 hours of incubation
**Yttrium-90 citrate colloid was administered on 24/07/2023**		
30/08/2023	18	-
27/09/2023	12	Turbid; no growth after 48 hours of incubation
17/10/2023	5	-
07/11/2023	5	-
12/12/2023	None	-
18/01/2024	None	-
28/02/2024	30	Purulent; numerous gram positive Bacilli seen; organism: Bacillus species

### Tumour volume

 There is a progressive shrinkage of the patient’s tumour volume following the irradiation therapy and the three months post therapy CECT brain demonstrated 94% reduction in tumour volume using the simple ellipsoidal volume calculation ( /6 • X • Y • Z). 

 The CECT brain (26/10/2023) showed a smaller cystic lesion at the suprasellar region with mild peripheral enhancement measuring 2.2×1.4×1.4 cm (previously 3.4×4.5×4.7 cm). 

**Figure 2:**
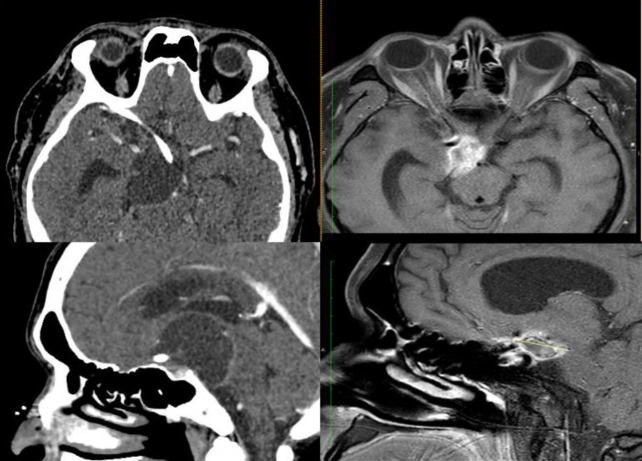
**A.** Pre therapy CECT (06/2023); **B.** Post therapy contrasted MRI (06/2024). Post therapy MRI demonstrated smaller suprasellar lesion with less mass effect onto the surrounding structures

 Magnetic resonance imaging (MRI) in the eleventh month of therapy (10/06/2024) demonstrated that the cystic lesion did not disappear completely but remained stable at the suprasellar region measuring 2.8×1.4×1.4 cm (Figure 2).

### Vision Function

 The patient is under ophthalmological follow up for right-eye blindness secondary to prolonged optic nerve compression with left temporal hemianopia. Following intracystic irradiation therapy, the patient reported improvement in the left visual field, along with fewer episodes of imbalance gait.

**Figure 3:**
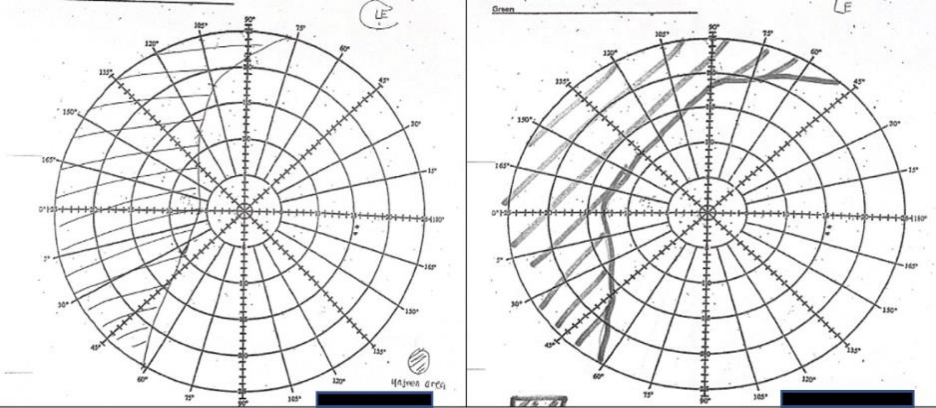
Pre and post therapy Bjerrum’s screen chart vision assessment, performed on 13/07/2023 and 13/11/2023 respectively. There was slightly reduction of scotoma at the central and superior part of the left eye. Visual acuity of left eye: 6/9 and right eye: No pupillary response

 These improvements are aligned with the Bjerrum’s screen chart findings, which revealed a slight reduction in scotoma in the central and superior regions of the left eye (Figure 3). However, the visual acuity in the left eye (6/9) and the right eye (no pupillary response) remained unchanged post-therapy.

### Hormonal Stability

 Routine hormonal blood investigations were taken and evaluated by the endocrinologist during the outpatient clinic follow up. No significant changes were found post therapy thus similar doses of hormonal replacement therapy were prescribed for the patient throughout the year. 

### Complications

 The patient did not experience any acute complications during the procedure. However, two weeks following treatment, the patient reported persistent nausea and vomiting, accompanied by severe headaches. He was subsequently readmitted and managed for a post-therapy inflammatory reaction, with his symptoms resolving promptly after the initiation of dexamethasone.

 Despite favourable outcomes during the initial six months, the patient's condition began to deteriorate by the seventh month post-therapy, and he presented to the hospital with severe headaches and behavioural changes. Immediate CSF aspiration yielded 30 mL of purulent fluid, and culture and sensitivity testing revealed the presence of numerous Gram-positive bacilli. Following a multidisciplinary discussion, it was determined that removal of the Ommaya reservoir was necessary, and the patient was treated with a three-week course of vancomycin. During hospitalisation, the patient was noted to develop right-sided ptosis. However, an urgent CT brain demonstrated no significant findings attributed to this new complaint and was thus treated conservatively. His right ptosis showed slight improvement upon discharge.

 Since discharge, it was reported that the patient did not require any further CSF aspiration. The most recent MRI (10/06/2024) indicated that the tumour remained stable in size. He no longer experienced any symptoms of increased intracranial pressure (i.e. nausea, vomiting and headache) and his hormonal blood investigation was within his baseline with no significant changes in hormonal medical replacement therapy. However, the patient continued to exhibit persistent two-thirds right-sided ptosis. Visual assessment demonstrated unchanged visual acuity as before therapy with 6/9 vision in the left eye and absent pupillary response in the right eye. It was also reported by the patient's carer that since discharge for the infected CSF at the seventh month post therapy, the patient had slowly developed a new onset of short-term memory loss. He had trouble remembering his daily activities (i.e. meal time and self-care) and thus hindered him from complete self-independency. He also has increased in appetite with a weight gain of 7 kg in one month. On further assessment, he scored 23 out of 30 on his mini mental state examination (MMSE) although no baseline MMSE was conducted prior to the irradiation therapy.

## Discussion

 Treatment strategies for craniopharyngioma focus on achieving tumour control while reducing morbidity. Therefore, the emphasis remains on developing individualised treatment plans that balance effective tumour management with the preservation of long-term patient well-being. Our patient exhibited a marked reduction in CSF aspiration frequency within the first month post-therapy, accompanied by a 94% decrease in tumour volume by three months, as shown on CECT imaging. By eight months, CSF aspiration was no longer required, correlating with stable tumour size on MRI. These results align with Pollock et al., who reported significant (>50%) volume reduction or complete resolution in 88% of cysts treated with phosphorus-32 intra-cavitary irradiation (7). Similarly, studies by Julow et al. and Vanhauwert et al. have demonstrated significant tumour volume reduction following Yttrium-90 irradiation in cystic craniopharyngioma patients (8, 9). According to Julow et al., who studied 78 patients, nearly 50% of the patients showed complete disappearance of the tumour in one-year duration post therapy (10).

 Previous studies have reported mixed outcomes in visual function following irradiation therapy, with prognosis largely dependent on baseline visual function. Cases with an intact optic disc or mild temporal pallor tend to show better visual recovery post-therapy. A study by Julow et al. reported vision improvement in 15 of 35 patients with intact optic discs or mild temporal pallor, compared to only 3 of 37 patients with severe pallor or atrophy. In our patient, the baseline visual function prior to therapy was poor, with no pupillary response in the right eye and 6/9 visual acuity in the left eye (10). Post-therapy, there was no significant change in visual acuity, though a slight improvement in the central left visual field scotoma was observed. The absence of significant visual recovery in our patient could be due to late-stage intervention with this irradiation therapy.

 Our patient successfully achieved excellent tumour control with the slight improvement of visual field and stable hormonal level. The functional outcome of our patient during the first sixth month was excellent, with fewer hospital visits and better daily living activities. 

 However, few complications were found to be associated, such as the subacute complication of posttherapy inflammatory changes that occurred at the second week posttherapy, which could be avoided by prophylactic anti-inflammatory drugs. Later complications of infected CSF requiring admission occurring at seven months post-therapy contributed to the patient's overall clinical deterioration and could potentially have been prevented by maintaining strict aseptic techniques during the monthly CSF aspiration procedures. He experienced residual right ptosis upon discharge, and this could be due to an injury to the right oculomotor nerve. This could probably result from either optic neuropathy related to the intracavitary irradiation therapy, traction on the optic apparatus as the cyst regresses, or secondary to the prolonged infected CSF. Since discharge home, the patient has also exhibited progressive symptoms indicative of hypo-thalamic dysfunction. Julow et al., in their study, have highlighted that beta irradiation effectively targets a 3–4 mm region outside the cyst wall, which could induce adverse reactions depending on its anatomical proximity (10). 

 This carries the possibility of radionecrosis to the hypothalamic region, including the fornix damage (1.3%) in patients with suprasellar cystic craniopharyngioma whom treated with intracystic irradiation therapy (10). Damage to the hypothalamic nuclei often manifests as hormonal disturbances, including hyperphagia, sleep disorders, and, in severe cases, mortality due to the hypothalamic dysfunction. Fornix injury may result in cognitive impairments, such as memory dysfunction or persistent memory loss, including Korsakoff’s syndrome (10). Despite the absence of baseline MMSE to objectively assess our patient, his current mental state and other hypothalamic symptoms raise the possibility of cognitive dysfunction as the complication of intracystic irradiation therapy.

 In addition to radionecrosis of the pituitary gland, hypothalamus, and anterior fornix, other complications of intracystic irradiation that warrant careful monitoring include radio-necrosis of the carotid artery and the pontomesencephalothalamic region (10). 

 Radiation exposure through the thin walls of retrosellar and interpeduncular cysts may damage the surrounding arterial branches of the vertebrobasilar system, potentially leading to clinical manifestations such as paramedian thalamic or peduncular pontine infarcts or stroke (10). In addition to a thorough assessment via MRI to examine the chiasma, fornix, and interpeduncular perforating arteries prior to therapy, a different approach of multiple lower radiation doses to the tumour could be an option to minimise radiation effects on the surrounding structures. Multiple studies have suggested different radiation doses in the range of 50–1000 Gy to the tumour lining (10). 

 Other options of radiopharmaceuticals with lower maximum energy and tissue penetration (i.e., phosphorus-32) could also be an option to reduce radiation exposure to the surrounding structures.

 Craniopharyngiomas present a significant therapeutic challenge due to their rarity, critical anatomical location, and diverse range of presenting symptoms. Treatment approaches for this benign tumour extend beyond intracavitary irradiation therapy and include surgery, stereotactic radiosurgery, conventional radiotherapy, proton therapy, chemotherapy, and systemic therapy. In this case, the patient underwent two resection surgeries prior to the decision to insert an Ommaya shunt, prompted by the tumour's persistent recurrence. Radiotherapy was deemed unsuitable due to the tumour's proximity to the brainstem. Modern radiotherapy techniques, particularly fractionated radiotherapy with doses ranging from 50 to 60 Gy, utilising CT-based treatment planning with or without MRI fusion, have demonstrated high tumour control rates of 92% to 100% (11). 

 However, previous studies suggest that patients receiving radiotherapy for recurrent craniopharyngiomas may experience less favourable outcomes compared to those treated with immediate adjuvant radiotherapy (11).

 The most critical indicator of a successful management strategy for craniopharyngioma is the patient’s ability to maintain independent function following therapy. In our case, the follow up demonstrated a valuable reduction in tumour volume, along with improved visual function and stable hormone levels post treatment. However, the complications including the cognitive dysfunction observed in our patient highlight the need for cautious consideration of intra-cavitary irradiation therapy, which should be reserved as a later option after a comprehensive pre-treatment evaluation.

## Data Availability

All data and material are available in the institutional archive system and included in this published article.

## References

[1] Nothfield DC (.1957). Rathke-pouch Tumours. Brain.

[2] Yaşargil MG, Curcic M, Kis M, Siegenthaler G, Teddy PJ, Roth P (1990). Total removal of craniopharyngiomas. Journal of Neuro-surgery..

[3] Fischer EG, Welch K, Shillito J, Winston KR, Tarbell NJ (1990). Long-term effects of conservative surgical procedures combined with radiation therapy. Journal of Neurosurgery..

[4] Bartels U, Laperriere N, Bouffet E, James D (2012). Intracystic therapies for cystic cranio-pharyngioma in childhood. Frontiers in Endocrinology Pituitary Endocrinology..

[5] Derrey S, Blond S, Reyns N, Touzet G, Carpentier P, Gauthier H (2008). Management of cystic craniopharyngiomas with stereotactic endocavitary irradiation using colloidal 186Re: a retrospective study of 48 consecutive patients. Neurosurgery..

[6] Abd Razak N, Pung CP, Kamarulzaman K, Amir Hassan SZ (2024). First local experience of intra-cavitary Yttrium-90 citrate colloid irradiation via ommaya reservoir for refractory cystic craniopharyngioma: a case report. Journal Nuclear Medicine and Molecular Imaging..

[7] Pollock BE, Lunsford LD, Kondziolka D, Levine G, Flickinger AJC (1995). Phosphorus-32 intracavitary irradiation of cystic craniopharyngiomas: current technique and long term results. International Journal of Radiation Oncology, Biology, Physics..

[8] Julow JV (2013). Intracystic irradiation for craniopharyngiomas. Pituitary..

[9] Vanhauwaert D, Hallaert G, Baert E, Van Roost D, Okito JPK, Caemaert J (2013). Treatment of cystic craniopharyngioma by endo-cavitary instillation of Yttrium-90 radioisotope - still a valuable treatment option. Journal of Neurological Surgery Part A: Central European Neurosurgery..

[10] Julow J, Backlund EO, Lányi F, Hajda M, Bálint K, Nyáry I (2007). Long-term results and late complications after intracavitary yttrium-90 colloid irradiation of recurrent cystic craniopharyngiomas. Neurosurgery..

[11] Varlotto J, DiMaio C, Grassberger C, Tangel M, Mackley H, Pavelic M (2015). Multi-modality management of cranio-pharyngioma: a review of various treatments and their outcomes. Neuro-Oncology Practice..

